# The impact of maternal prenatal psychological distress on the development of epilepsy in offspring: The Japan Environment and Children’s Study

**DOI:** 10.1371/journal.pone.0311666

**Published:** 2024-11-13

**Authors:** Yuto Arai, Tohru Okanishi, Toshio Masumoto, Hisashi Noma, Yoshihiro Maegaki, on behalf of the Japan Environment and Children’s Study Group

**Affiliations:** 1 Faculty of Medicine, Department of Brain and Neurosciences, Division of Child Neurology, Tottori University, Yonago, Japan; 2 Faculty of Medicine, Department of Social Medicine, Division of Health Administration and Promotion, Tottori University, Yonago, Japan; 3 Department of Data Science, The Institute of Statistical Mathematics, Tokyo, Japan; Injibara University, ETHIOPIA

## Abstract

The relationship between maternal prenatal psychological distress and epilepsy development in offspring has not yet been clarified. Herein, we used a dataset obtained from the Japan Environment and Children’s Study, a nationwide birth cohort study, to evaluate the association between six-item Kessler Psychological Distress Scale (K6) scores and epilepsy among 1–3 years old. The data of 97,484 children were retrospectively analyzed. The K6 was administered to women twice: during the first half (M-T1) and second half (M-T2) of pregnancy. M-T1 ranged from 12.3–18.9 (median 15.1) weeks, and M-T2 ranged from 25.3–30.1 (median 27.4) weeks. Participants were divided into six groups based on K6 scores of two ranges (≤4 and ≥5) at M-T1 and M-T2. The numbers of children diagnosed with epilepsy at the ages of 1, 2, and 3 years were 89 (0.1%), 129 (0.2%), and 149 (0.2%), respectively. A maternal K6 score of ≥5 at both M-T1 and M-T2 was associated with epilepsy diagnosis ratios among 1-, 2-, and 3-year-old children in the univariate analysis. Moreover, multivariate analysis revealed that a maternal K6 score of ≥5 at both M-T1 and M-T2 was associated with epilepsy diagnosis ratios among 1-, 2-, and 3-year-olds. Continuous moderate-level maternal psychological distress from the first to the second half of pregnancy is associated with epilepsy among 1-, 2-, and 3-year-old children. Hence, environmental adjustments to promote relaxation such as mindfulness in pregnant women might be necessary.

## Introduction

Epilepsy, affecting 65 million individuals globally, is the most prevalent, persistent, and severe neurological disorder worldwide [1]. Individuals living with epilepsy often encounter discrimination, misunderstanding, and social stigma [2], and they endure the stress of living with a chronic, unpredictable disease that may lead to a loss of autonomy in activities of daily living. Epilepsy is one of the most common neurological disorders in childhood [3], while epilepsy onset before the age of three is particularly associated with high rates of drug resistance and developmental delays [4]. As such, it is crucial to develop preventive measures to reduce the incidence of epilepsy in children under the age of three. Previous studies examining big data on the perinatal environment have identified abruptio placenta, eclampsia, infection in pregnancy, low birth weight, and artificial milk feeding as risk factors for the onset of early childhood epilepsy [5].

The fetal programming theory proposes that the environment status during fetal development significantly impacts health and disease risk throughout an individual’s lifetime [6–10]. This theory underscores the critical importance of the fetal environment, providing a novel perspective in preventive medicine by highlighting its significant impact on the future health of children. Studies using animal models have shown that maternal distress can influence the development of the fetal nervous system and the function of the hypothalamic-pituitary-adrenal (HPA) axis, leading to long-term negative effects on the offspring’s learning, motor development, and behavior [11–14]. Previous research has demonstrated that maternal psychological stress can lead to neuropsychological disorders in children, such as anxiety, depression, and attention deficit hyperactivity disorder [15,16]. On the other hand, epilepsy is a neurological complication whose association with prenatal stress has not yet been fully established. However, some reports have suggested that prenatal distress may alter the glutamatergic [17,18], gamma-aminobutyric acidergic [19,20], and adrenergic systems [21,22], which are closely related to seizures in epilepsy patients, through the HPA axis, indicating a potential link to the onset of epilepsy. Moreover, while the pathophysiology of epilepsy results from abnormalities in brain networks [23], the synthesis of glucocorticoids and release of cytokines induced by prenatal maternal stress influence the programming of the functional structure and connectivity of the offspring’s brain [24–28]. Therefore, maternal stress during pregnancy may be directly or indirectly associated with the development of epilepsy in children.

In Japan, identified concerns include shortened maternal sleep duration during pregnancy [29] and prolonged working hours [30]. From the perspective of the fetal programming theory, these environmental factors are believed to exacerbate maternal stress and psychological burden, thus potentially intensifying the neurological impacts on the fetus. If prenatal stress is found to be associated with the onset of childhood epilepsy, then addressing and improving such environmental factors would become a necessity.

The Japan Environment and Children’s Study (JECS) is a nationwide government-funded birth cohort study conducted by the Japanese Ministry of the Environment. This extensive national study encompasses 100,000 sets of parents and children and is designed to explore the relationships between environmental factors and child development [31,32]. Within this vast dataset, various perinatal environmental factors, including maternal psychological stress, and outcome factors, including epilepsy, were recorded. We hypothesized that prenatal maternal stress influences the onset of epilepsy in children and aimed to investigate the hypothesis using the JECS data.

## Material and methods

### Design and participants

This study utilized the JECS dataset to retrospectively investigate the relationship between maternal prenatal psychological distress and epilepsy in children aged 1, 2, and 3 years.

The JECS methodology has been detailed in past publications [31,32]. The JECS recruited pregnant women from across the nation from January 2011 to March 2014. The study is ongoing, with plans to continue monitoring participants until the children reach the age of 13 years. As inclusion criteria, we utilized all 104,062 mother-child pairs from the jeсs-ta-20190930 dataset, which was released in October 2019 and revised in 2022. Cases involving abortion or stillbirth, as well as those with missing data on the six-item Kessler Psychological Distress Scale (K6), were excluded.

### Maternal psychological distress

The JECS protocol was structured to facilitate the administration of K6 on two occasions during pregnancy: in the first (M-T1) and second (M-T2) halves of gestation [32]. The K6 is a self-administered psychological distress scale commonly utilized in population-based research and primary healthcare settings [33–35]. The six items of the K6 (feeling nervous, hopeless, restless or fidgety, worthless, sad, and that everything is an effort) were grouped into depressive and anxiety symptoms. This was based on the Diagnostic and Statistical Manual of Mental Disorders, Fourth Edition, reflecting the preceding four weeks on a scale of 0 to 4 [36]. Previous reports have demonstrated the efficacy of the K6 as a screening scale to detect depressive and anxiety disorders [33]. The total score is the sum of the six items and ranges from 0 to 24, with a cutoff ≥5 to identify cases of moderate-level psychological distress [37]. We used the Japanese version of the K6 with a cutoff ≥5, which has been used in previous studies involving the general population and specific communities in Japan [38–40].

The progression of fetal development differs between the early and mid/late stages of pregnancy [41]. In the early stages, major organs and structures such as the brain, heart, and limbs develop. Subsequently, from the mid to late stages, myelination and the formation of brain grooves occur, establishing neural networks within the brain. Given that the impact of stress on the fetus may vary depending on the timing and the specific organs affected, we classified the participants into six groups based on their maternal K6 scores at M-T1 and M-T2: (1) K6 scores of ≥5 at M-T1; (2) K6 scores of ≥5 at M-T2; (3) K6 scores of ≤4 at M-T1 and M-T2; (4) K6 scores of ≥5 at M-T1 and ≤4 at M-T2; (5) K6 scores of ≤4 at M-T1 and ≥5 at M-T2; and (6) K6 scores of ≥5 at M-T1 and M-T2.

### Outcome: Epilepsy among 1-, 2-, and 3-year-old children

Based on data obtained from the C-1-, -2-, and -3-year questionnaires (performed when the child was 1, 2, and 3 years of age, respectively), we estimated the incidence of epilepsy each year. In the self-reported questionnaire, mothers were asked, “Has your child been diagnosed with epilepsy by a physician?”. Children whose mothers answered “Yes” were defined as having a diagnosis of epilepsy.

### Data collection of potential prognostic factors

We collected data about potential prognostic factors associated with neurodevelopment from the questionnaires, including maternal academic and social history [42,43], history of maternal neuropsychiatric disorders [44,45], psychoactive drug use during pregnancy [46], maternal epilepsy [47], maternal alcohol consumption during pregnancy [48], hypertensive disorders complicating pregnancy and labor abruption, premature birth of children, low birth weight, chromosome abnormalities, and nutrition at 1 month of age [5].

### Statistical analyses and covariables

Data analysis was performed to determine the association between K6 scores of ≥5 and epilepsy among 1-, 2-, and 3-year-old children. First, we performed univariate logistic regression analyses to evaluate potential prognostic factors for K6 score variables individually. Subsequently, we performed multivariate logistic regression analyses to adjust potential confounding biases involving factors detected in the univariate analyses (*p*<0.05). Multicollinearity occurs when variables are highly correlated, making it difficult to precisely identify the extent to which each variable influences the dependent variable. Therefore, we further assessed multicollinearity through multivariate analysis. The variance inflation factor (VIF) was used to assess for multicollinearity in multivariate regression models. VIF>10 was defined as serious multicollinearity. Data analysis was performed using IBM SPSS Statistics version 25.0 (IBM Japan, Tokyo, Japan).

### Ethical approval

This study was conducted according to the guidelines of the Declaration of Helsinki. The JECS protocol was reviewed and approved by the Ministry of the Environment’s Institutional Review Board on Epidemiological Studies (no. 100910001) and by the Ethics Committees of all participating institutions. Written informed consent was obtained from all participating mothers and fathers. Children for whom parental written consent could not be obtained were excluded from the study.

## Results

### Participants

**Fig 1:**
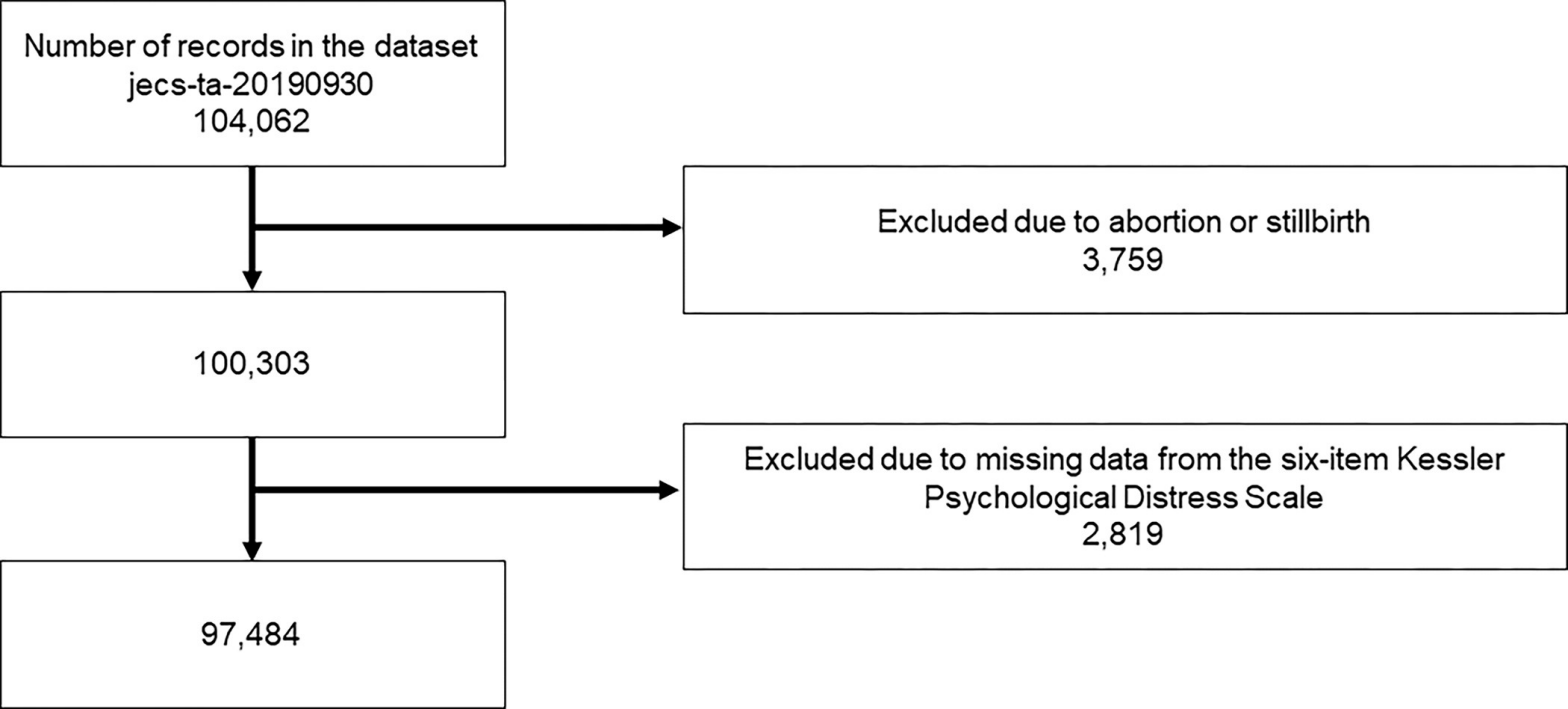
Flow chart showing the selection process of study participants.

Of the 104,062 entries in the dataset, 3,759 in which pregnancies with miscarriages or stillbirths occurred and 2,819 with missing K6 scale data for either the M-T1 or M-T2 were excluded. As a result, data from 97,484 children were analyzed (Fig 1). The demographic information of the participants is presented in Table 1. At M-T1, the maternal prenatal K6 score was determined at a median of 15.1 (interquartile range 12.3–18.9) weeks of gestation. At M-T2, the maternal prenatal K6 score was assessed at a median of 27.4 (interquartile range 25.3–30.1) weeks of gestation.

**Table 1:** Characteristics of the study participants.

		Total	
		n = 97,484	
		n	%
Maternal age at delivery (years)	< 20	799	0.8
	20 to 24	8,654	8.9
	25 to 34	61,525	63.1
	≥ 35	26,498	27.2
	No answer	8	0.0
Paternal age at conception (years)	< 20	211	0.2
	20 to 24	3,205	3.3
	25 to 34	28,052	28.8
	≥ 35	18,165	18.6
	No answer	47,851	49.1
Maternal status	Married	92,930	95.3
	Divorced, lost, other	4,148	4.3
	No answer	406	0.4
Maternal academic history	College, university	60,348	61.9
	Senior high school	32,085	32.9
	Junior high school	4,657	4.8
	No answer	394	0.4
Paternal academic history	College, university	51,927	53.3
	Senior high school	37,537	38.5
	Junior high school	7,020	7.2
	No answer	1,000	1.0
Maternal job during pregnancy	No	46,799	48.0
	Yes	49,924	51.2
	No answer	761	0.8
Maternal smoking during pregnancy	No	92,302	94.7
	Yes	4,389	4.5
	No answer	793	0.8
Maternal alcohol consumption during pregnancy	No	93,981	96.4
	Yes	2,700	2.8
	No answer	803	0.8
Household income (10^3^ yen/year) during pregnancy	< 4000	36,499	37.4
	4000 to 5999	30,024	30.8
	≥ 6000	24,310	24.9
	No answer	6,651	6.8
Maternal epilepsy	No	96,933	99.4
	Yes	551	0.6
Maternal neuropsychiatric disorder	No	89,971	92.3
	Yes	7,513	7.7
Psychoactive drug use during pregnancy	No	96,365	98.9
	Yes	1,119	1.1
Hypertensive disorders of pregnancy complication	No	96,301	98.8
	Yes	950	1.0
	No answer	233	0.2
Labor abruption	No	96,864	99.4
	Yes	387	0.4
	No answer	233	0.2
Sex of children	Male	47,515	48.7
	Female	49,959	51.2
	Unknown	2	0.0
	No answer	8	0.0
Birth weight of children (g)	< 1500	600	0.6
	1500 to 2499	8,243	8.5
	2500 to 3999	87,514	89.8
	≥ 4000	833	0.9
	No answer	294	0.3
Chromosome abnormalities of children	No	96,534	99.4
	Yes	189	0.2
	No answer	761	0.8
Gestation week	< 28	150	0.2
	28 to 33	918	0.9
	34 to 36	4,170	4.3
	37 to 41	91,754	94.1
	≥ 42	222	0.2
	No answer	270	0.3
Nutrition at 1 month of age	Breast milk	40,141	41.2
	Breast milk or milk	54,223	55.6
	Milk	1,417	1.5
	No answer	1,703	1.7
Maternal K6 scores	M-T1; K6 ≥ 5	31,075	31.9
	M-T2; K6 ≥ 5	28,124	28.8
	M-T1; K6 ≤ 4 and M-T2; K6 ≤ 4	56,512	58.0
	M-T1; K6 ≥ 5 and M-T2; K6 ≤ 4	12,848	13.2
	M-T1; K6 ≤ 4 and M-T2; K6 ≥ 5	9,897	10.2
	M-T1; K6 ≥ 5 and M-T2; K6 ≥ 5	18,227	18.7
Epilepsy	1 year (n = 88,984)	89	0.1
	2 years (n = 84,724)	129	0.2
	3 years (n = 81,190)	149	0.2

*K6*, 6-item Kessler Psychological Distress Scale; *M-T1*, median 15.1 (12.3–18.9) weeks of gestation; *M-T2*, median 27.4 (25.3–30.1) weeks of gestation.

The means of K6 scores during M-T1 and the M-T2 were 3.6 and 3.5, respectively. The numbers (ratios) of the datasets in the six groups were as follows: (1) K6 scores ≥5 at M-T1: 31,075 mothers (31.9%); (2) K6 scores ≥5 at M-T2: 28,124 (28.8%); (3) K6 scores ≤4 at M-T1 and M-T2: 56,512 mothers (58.0%); (4) K6 scores ≥5 at M-T1 and ≤4 at M-T2: 12,848 (13.2%); (5) K6 scores ≤4 at M-T1 and ≥5 at M-T2: 9,897 (10.2%); and (6) K6 scores ≥5 at M-T1 and M-T2: 18,227 (18.7%). Children diagnosed with epilepsy at the ages of 1, 2, and 3 years were 89 (0.1%), 129 (0.2%), and 149 (0.2%), respectively.

### Statistical results

#### Univariate analysis

Table 2 presents the results of univariate logistic regression analyses for maternal K6 score and epilepsy of 1-, 2-, and 3-year-old children. The analyses revealed that a maternal K6 scores of ≥5 at M-T1 was not associated with epilepsy among 1- (odds ratio [OR]: 1.4, 95% confidence interval [CI], 0.92–2.2, *p* = 0.11), 2- (OR: 1.2, 95% CI, 0.85–1.8, *p* = 0.29) and 3-year-old children (OR: 1.1, 95% CI, 0.8–1.6, *p* = 0.47). A maternal K6 scores of ≥5 at M-T2 was not associated with epilepsy among 1- (OR: 1.5, 95% CI, 0.98–2.3, *p* = 0.063), 2- (OR: 1.3, 95% CI, 0.86–1.8, *p* = 0.24) and 3-year-old children (OR: 1.4, 95% CI, 0.95–1.9, *p* = 0.092). K6 scores ≤4 at M-T1 and M-T2 was not associated with epilepsy among 1- (OR: 0.73, 95% CI, 0.48–1.1, *p* = 0.13), 2- (OR: 0.97, 95% CI, 0.68–1.4, *p* = 0.86) and 3-year-old children (OR: 0.95, 95% CI, 0.68–1.3, *p* = 0.78). K6 scores ≥5 at M-T1 and ≤4 at M-T2 was not associated with epilepsy among 1- (OR: 0.92, 95% CI, 0.49–1.7, *p* = 0.79), 2- (OR: 0.68, 95% CI, 0.38–1.2, *p* = 0.21) and 3-year-old children (OR: 0.57, 95% CI, 0.31–1.1, *p* = 0.74). K6 scores ≤4 at M-T1 and ≥5 at M-T2 was not associated with epilepsy among 1- (OR: 1.01, 95% CI, 0.51–2.0, *p* = 0.97), 2- (OR: 0.61, 95% CI, 0.30–1.3, *p* = 0.18) and 3-year-old children (OR: 0.81, 95% CI, 0.44–1.5, *p* = 0.51). However, a maternal K6 score of ≥5 at both M-T1 and M-T2 was associated with epilepsy among 1- (OR: 1.7, 95% CI, 1.04–2.7, *p* = 0.033), 2- (OR: 1.6, 95% CI, 1.1–2.4, *p* = 0.017) and 3-year-old children (OR: 1.6, 95% CI, 1.1–2.4, *p* = 0.014).

**Table 2:** Association between the development of epilepsy in children at 1-, 2-, and 3-years of age and maternal K6 score based on univariate logistic regression analyses.

Maternal K6 score	With epilepsy (1 year) n = 89			With epilepsy (2 years) n = 129			With epilepsy (3 years) n = 149		
	OR	95% CI	*p*-value	OR	95% CI	*p*-value	OR	95% CI	*p*-value
M-T1; K6 ≥ 5	1.4	0.92–2.2	0.11	1.2	0.85–1.8	0.29	1.1	0.80–1.6	0.47
M-T2; K6 ≥ 5	1.5	0.98–2.3	0.063	1.3	0.86–1.8	0.24	1.4	0.95–1.9	0.092
M-T1; K6 ≤ 4 and M-T2; K6 ≤ 4	0.73	0.48–1.1	0.13	0.97	0.68–1.4	0.86	0.95	0.68–1.3	0.78
M-T1; K6 ≥ 5 and M-T2; K6 ≤ 4	0.92	0.49–1.7	0.79	0.68	0.38–1.2	0.21	0.57	0.31–1.1	0.074
M-T1; K6 ≤ 4 and M-T2; K6 ≥ 5	1.01	0.51–2.0	0.97	0.61	0.30–1.3	0.18	0.81	0.44–1.5	0.51
M-T1; K6 ≥ 5 and M-T2; K6 ≥ 5	1.7	1.04–2.7	0.033*	1.6	1.1–2.4	0.017*	1.6	1.1–2.4	0.014*

*CI*, confidence interval; *K6*, 6-item Kessler Psychological Distress Scale; *M-T1*, median 15.1 (12.3–18.9) weeks of gestation; *M-T2*, median 27.4 (25.3–30.1) weeks of gestation; *OR*, odds ratio

*p<0.05.

#### Multivariate analyses

Table 3 presents the results of the multivariate logistic regression analysis for a maternal K6 score of ≥5 at M-T1 and M-T2 and epilepsy of 1-, 2-, and 3-year-old children. As the covariates for the analysis, potential prognostic factors and a maternal K6 score of ≥5 at both M-T1 and M-T2, which were associated with epilepsy in the univariate logistic regression analysis (Table 2), were included. The analysis demonstrated that a maternal K6 score of ≥5 at both M-T1 and M-T2 was associated with epilepsy among 1- (OR: 1.7, 95% CI, 1.1–2.9, *p* = 0.030), 2- (OR: 1.7, 95% CI, 1.1–2.6, *p* = 0.012) and 3-year-old children (OR: 1.7, 95% CI, 1.1–2.6, *p* = 0.012). Moreover, birth weight <2500 g was associated with epilepsy in 1-year-olds (OR: 2.8, 95% CI, 1.4–4.7, *p* = 0.005), and nutrition with artificial milk at 1 month of age was associated with epilepsy in 2-year-olds (OR: 3.03, 95% CI, 1.2–7.6, *p* = 0.022). Additionally, chromosome abnormalities were associated with epilepsy in 1- (OR: 22.0, 95% CI, 7.3–79.2, *p*<0.001), 2- (OR: 16.7, 95% CI, 5.1–54.6, *p*<0.001), and 3-year-olds (OR: 11.0, 95% CI, 2.6–44.6, *p* = 0.001). Among epilepsy in 1-, 2- and 3-year-old children, the VIF of maternal academic history: junior high school, maternal alcohol consumption during pregnancy, household income during pregnancy: <4000 yen/year, maternal epilepsy, maternal neuropsychiatric disorder, psychoactive drug use during pregnancy, hypertensive disorders of pregnancy complication, labor abruption, gestation week: <37, birth weight of children: <2500 g, chromosome abnormalities, nutrition at 1 month of age: milk, and M-T1; K6 ≥ 5 and M-T2; were <10 respectively, indicating no multicollinearity among these variables.

**Table 3:** Association between the development of epilepsy in children at 1-, 2-, and 3-years of age and maternal K6 score based on multivariate logistic regression analyses.

Maternal K6 score	With epilepsy (1 year) n = 89				With epilepsy (2 years) n = 129				With epilepsy (3 years) n = 149			
	OR	95% CI	*p*-value	VIF	OR	95% CI	*p*-value	VIF	OR	95% CI	*p*-value	VIF
Maternal academic history: junior high school	1.9	0.84–4.2	0.11	1.06	1.01	0.41–2.5	0.88	1.03	1.2	0.53–2.8	0.63	1.04
Maternal alcohol consumption during pregnancy	0.84	0.21–3.4	0.78	1.00	1.9	0.82–4.3	0.14	1.00	2.1	0.97–4.5	0.059	1.00
Household income during pregnancy: <4000 yen/year	1.6	0.99–2.4	0.052	1.04	1.1	0.74–1.6	0.72	1.03	0.98	0.68–1.4	0.91	1.03
Maternal Epilepsy	0	–	0.98	1.00	1.4	0.18–11.1	0.73	1.09	1.2	0.16–9.5	0.84	1.07
Maternal neuropsychiatric disorder	0.65	0.27–1.6	0.35	1.17	0.85	0.42–1.7	0.66	1.09	1.2	0.66–2.2	0.53	1.13
Psychoactive drug use during pregnancy	2.2	0.47–9.8	0.32	1.17	1.1	0.24–5.1	0.89	1.18	1.5	0.43–5.2	0.53	1.20
Hypertensive disorders of pregnancy complication	0.92	0.13–7.3	0.94	1.03	0.74	0.1–5.4	0.76	1.02	0.75	0.10–5.5	0.77	1.02
Labor abruption	2.7	0.37–20.5	0.32	1.02	2.02	0.27–14.5	0.51	1.02	4.1	0.96–16.7	0.058	1.04
Gestation week: <37	0.52	0.22–1.6	0.2	1.25	0.89	0.39–2.0	0.76	1.33	1.02	0.44–2.4	0.96	1.35
Birth weight of children: <2500 g	2.8	1.4–4.7	0.005*	1.24	1.8	0.97–3.2	0.063	1.32	1.3	0.65–2.4	0.5	1.34
Chromosome abnormalities	22.0	7.3–79.2	<0.001*	1.03	16.7	5.1–54.6	<0.001*	1.03	11.0	2.6–44.6	0.001*	1.02
Nutrition at 1 month of age: milk	2.1	0.65–6.9	0.22	1.05	3.03	1.2–7.6	0.022*	1.06	2.3	0.79–6.2	0.13	1.06
M-T1; K6 ≥ 5 and M-T2; K6 ≥ 5	1.7	1.1–2.9	0.030*	1.05	1.7	1.1–2.6	0.012*	1.04	1.7	1.1–2.6	0.012*	1.05

*CI*, confidence interval; *K6*, 6-item Kessler Psychological Distress Scale; *M-T1*, median 15.1 (12.3–18.9) weeks of gestation; *M-T2*, median 27.4 (25.3–30.1) weeks of gestation; *OR*, odds ratio

*p<0.05; VIF, variance inflation factor.

## Discussion

Fetal programming theory posits that environmental factors, such as stress during pregnancy, can have long-term effects on fetal development [6–10]. Recent reports have increasingly indicated that prenatal stress can impact the central nervous system of the offspring in human study [27,40,49,50]. Consequently, we investigated the correlation between prenatal stress and the onset of epilepsy in offspring. The JECS was based on self-reported data from participants; however, the incidence of epilepsy up to age three was similar to data from the United Kingdom [4]. This suggests that our data is generally comprehensive for developed countries. As a result, this study demonstrated that continuous moderate-level maternal psychological distress from the first to the second half of pregnancy was associated with an increased risk of epilepsy among 1-, 2-, and 3-year-old children. Furthermore, nutrition with artificial milk, low birth weight, and chromosomal abnormalities were identified as risk factors.

Prenatal stress is associated with the onset of epilepsy in children. Our findings support previous reports indicating that prenatal stress increases the risk of neurological disorders in offspring and affirm the fetal programming theory [6–10,40,51]. Significant stress during pregnancy may potentially affect epileptogenesis through mechanisms such as changes in the DNA and histone methylation [52]. Moreover, both animal and human studies have revealed that prenatal exposure to maternal anxiety or depression is associated with changes in fetal brain structure and function, especially in the prefrontal cortex, hippocampus, and amygdala [24–28,53,54]. Epilepsy is caused by the generation of abnormal networks, and certain types of epilepsy exhibit network abnormalities in these areas [23,55–57]. Brain networks develop over several years after birth [58]. As such, these factors are associated with the development of epilepsy in children under 3 years of age.

Artificial milk nutrition has been associated with epilepsy in 2-year-olds. Previous reports indicate that breastfeeding may prevent the onset of epilepsy in children [5,59], and our findings are consistent with these reports. Breast milk contains various bioactive agents required for optimal infant brain development, such as arachidonic acid (AA) and docosahexaenoic acid (DHA) [60]. During the last trimester and neonatal period, brain tissue is rapidly synthesized, and cell differentiation and development of active synapses in the brain have specific requirements for DHA and AA [60]. The literature recommends the addition of AA and DHA to regular artificial milk as their levels do not meet those found in breast milk [61]. As such, the deficiency of essential components necessary for normal neurodevelopment in artificial milk may have resulted in an increased risk of developing epilepsy.

Low birth weight has been found to be associated with epilepsy in 1-year-olds, as previously reported [62]. The birth weight of children with epilepsy is lower than that of healthy controls [63]. Low birth weight is associated with brain structure abnormalities, including ventricular dilation, smaller brain volume, reduced cortical surface area, regional cortical thinning, and white matter abnormalities [64]. Low birth weight is also associated with network abnormalities in the hippocampus and amygdala [65]. Abnormal brain structure and function resulting from low birth weight have been considered to be associated with the onset of epilepsy.

Chromosome abnormalities were also found to be associated with epilepsy in 1-, 2-, and 3-year-olds. Approximately 400 chromosomal imbalances associated with seizures or EEG abnormalities have been reported [66]. Some chromosomal disorders are strongly associated with the onset of epilepsy throughout childhood [66,67]. However, due to the lack of detailed chromosomal information, we were unable to determine which specific chromosomal abnormalities contributed to the onset of childhood epilepsy in this study.

Preventing the onset of epilepsy is necessary as this condition is strongly associated with psychological and social problems in everyday life [2]. Concerning artificial milk nutrition and low birth weight, nutrition guidance during pregnancy, avoidance of smoking, and recommendation of breastfeeding may be effective [60,68]. Moreover, relaxation therapies, including yoga, music, Benson therapy, progressive muscle relaxation, deep breathing relaxation, guided imagery, mindfulness, and hypnosis have been shown to reduce maternal stress and anxiety and alleviate depressive symptoms [69–71]. As such, relaxation interventions for pregnant women may also be effective in preventing the onset of epilepsy in offspring. Furthermore, in Japan, interventions to dissolve the problems in sleep quality [72] and workplace environment [73] during pregnancy may be particularly important to alleviate stress in pregnant women.

This study has some limitations. First, we could not conduct a detailed evaluation of the types of epilepsy in the affected offspring. Moreover, the diagnosis of epilepsy was based on responses from parents via questionnaires, and cases where there was a lack of understanding of the diagnosis may not be included in the aggregate data. The detailed classification of epilepsy by medical doctors rather than non-medical personnel will be necessary in future research. Second, the presence of confounding factors could not be ruled out completely. We have confirmed that there was no confounding among the potential factors in this study based on the VIF analysis. However, other potential confounding factors, such as genetic or chromosomal abnormalities associated with both epilepsy and stress [66,74], as well as paternal mental status during pregnancy [75], require further investigation in future studies. Thirdly, it is crucial to acknowledge the limitations of the K6. The K6 assessment is based on the past 30 days and might not reflect the overall stress experienced during the first half and second half of pregnancy [33,37,76]. Additionally, it does not provide specific information on the types of stress or psychological disorders that the mother may have encountered [77]. Moreover, data collection on non-psychological stress factors during early pregnancy has been limited, preventing a comprehensive evaluation.

In conclusion, the present study found that continuous moderate-level maternal psychological distress from the first to the second half of pregnancy was associated with epilepsy among 1-, 2-, and 3-year-old children. As previously reported, additional risk factors include artificial milk nutrition, low birth weight, and chromosomal abnormalities. Therefore, environmental adjustments to promote relaxation in pregnant women are needed to prevent the development of epilepsy in their offspring.
